# Influence of Aqueous, Ethanolic, and Hydroethanolic Extraction on Phytochemical Content and Cardiometabolic‐Related Bioactivities of Four Cameroonian Spices

**DOI:** 10.1155/bmri/1151249

**Published:** 2026-08-04

**Authors:** Kendra Lucrèce Nkaleu Ngonganga, Achille Nouga Bissoue, Blanche Cunégonde Omgba Etoundi, Aymar Rodrigue Fogang Mba, Ahlam Ali, Roberta Tolve, Fideline Laure Tchuenbou-Magaia, Fabrice Fabien Dongho Dongmo

**Affiliations:** ^1^ Laboratory of Process Engineering, Advanced Teacher′s Training College for Technical Education, University of Douala, Douala, Cameroon, univ-douala.com; ^2^ Department of Biochemistry, Faculty of Science, University of Douala, Douala, Cameroon, univ-douala.com; ^3^ Research Institute of Healthcare Sciences, School of Pharmacy and Life Sciences, Faculty of Science and Engineering, University of Wolverhampton, Wolverhampton, UK, wlv.ac.uk; ^4^ Department of Biotechnology, University of Verona, Verona, Italy, univr.it; ^5^ Centre for Engineering Innovation and Research, School of Architecture, Computing and Engineering, Faculty of Science and Engineering, University of Wolverhampton, Wolverhampton, UK, wlv.ac.uk

**Keywords:** antioxidants, cardiometabolic health, enzyme inhibition, extraction solvent, inflammation activity

## Abstract

**Background:**

Cameroonian spices are rich in bioactive phenolics with potential applications in functional foods and nutraceuticals. Yet, the influence of solvent polarity on phytochemical extraction and the resulting multifunctional in vitro activities remains insufficiently characterized. This study compared aqueous, ethanolic, and hydroethanolic extracts of *Mondia whitei*, *Scorodophloeus zenkeri*, *Syzygium aromaticum*, and *Xylopia aethiopica*, linking solvent‐dependent phytochemical profiles to antioxidant, metabolic, and anti‐inflammatory activities relevant to cardiometabolic health.

**Methods:**

Total polyphenols, flavonoids, and condensed tannins were quantified using standard colorimetric assays. Antioxidant capacity was assessed through the 2,2‐diphenyl‐1‐picrylhydrazyl (DPPH), 2,2 ^′^‐azino‐bis(3‐ethylbenzothiazoline‐6‐sulfonic acid) (ABTS), and ferric reducing antioxidant power (FRAP) assays. Enzyme inhibition was evaluated for *α*‐amylase, *α*‐glucosidase, *β*‐galactosidase, pancreatic lipase, 3‐hydroxy‐3‐methylglutaryl‐CoA reductase (HMG‐CoA reductase), angiotensin‐converting enzyme (ACE), cyclooxygenases (COX‐1 and COX‐2), and 5‐lipoxygenase (5‐LOX). Half‐maximal inhibitory concentrations (IC_50_) were determined from nonlinear regression of dose–response curves.

**Results:**

Solvent polarity markedly influenced extraction yield and phytochemical composition. Hydroethanolic extraction provided the most balanced recovery of phenolics and consistently enhanced antioxidant, metabolic, and anti‐inflammatory responses. Ethanolic extracts of *S. zenkeri* and *M. whitei* exhibited high polyphenol content and strong inhibition of HMG‐CoA reductase and ACE. *S. aromaticum* showed broad antioxidant and anti‐inflammatory activity, whereas *X. aethiopica* displayed moderate but reliable effects across assays. Correlation trends revealed selective associations between specific phytochemical classes and biological endpoints, indicating that bioactivity depends on both phytochemical composition and solvent‐driven extraction patterns.

**Conclusion:**

Hydroethanolic extraction generally yielded phenolic‐rich, multifunctional extracts across the four Cameroonian spices, although solvent performance remained species‐dependent. The combined solvent‐dependent trends and phytochemical–bioactivity correlations highlight that extraction polarity and phytochemical composition jointly shape the functional signatures of these spices. These findings provide preliminary biochemical indicators of their potential for functional food and phytomedicinal applications. Further work involving comprehensive chemical characterization and validation in cell‐based and in vivo models is required to clarify mechanisms of action and support future development pathways.

**Highlights**

• Hydroethanolic extraction enhanced the recovery of polyphenols, flavonoids, and tannins across all species.
• The ethanolic extract of *Scorodophloeus zenkeri* showed exceptionally strong angiotensin‐converting enzyme (ACE) and 3‐hydroxy‐3‐methylglutaryl‐coenzyme A reductase (HMG‐CoA reductase) inhibition under in vitro conditions.
• *Mondia whitei* demonstrated potent *α*‐amylase and HMG‐CoA reductase inhibitory activity.
• *Syzygium aromaticum* exhibited broad antioxidant and anti‐inflammatory effectiveness across assays.
• Cameroonian spices offer multifunctional bioactivities supporting cardiometabolic protection.

## 1. Introduction

Spices hold a central place in culinary traditions worldwide, valued not only for their sensory attributes but also for their long‐recognized medicinal properties [1]. In Africa, and particularly in Cameroon, they are deeply embedded in daily cuisine and traditional healing practices, where they are used to alleviate a wide range of ailments. Cameroon′s exceptional ecological diversity supports more than 40 spice species distributed across over 20 botanical families [2], including Zingiberaceae (*Aframomum melegueta*), Piperaceae (*Piper guineense*), Annonaceae (*Xylopia aethiopica*), Fabaceae (*S. zenkeri*), and Apocynaceae (*M. whitei*). These species are rich in polyphenols, flavonoids, tannins, and essential oils [3], natural compounds also found in fruits, vegetables, nuts, tea, and wine. Such molecules are widely associated with antioxidant, anti‐inflammatory, and antimicrobial activities [4, 5] and play a central role in mitigating oxidative stress, a major driver of chronic and infectious diseases [3, 6]. Despite their cultural and therapeutic importance, many Cameroonian spices remain insufficiently characterized at the phytochemical and pharmacological levels, underscoring the need for more systematic scientific investigation.

The biological value of spice‐derived bioactives depends strongly on extraction conditions. Solvent polarity, temperature, and extraction duration influence the recovery, stability, and functional efficacy of phenolic constituents [7–9]. Extraction techniques range from simple maceration, slow and prone to contamination [10], to infusion and decoction, which accelerate extraction but may degrade thermolabile compounds. More advanced methods such as ultrasound‐assisted extraction, Soxhlet, and supercritical CO_2_ can improve yields but require costly equipment and technical expertise [11], limiting their accessibility in low‐resource settings. In Cameroon, where culinary and medicinal practices traditionally rely on water‐ and alcohol‐based preparations, aqueous and ethanolic extractions remain the most practical and culturally aligned. Ethanol efficiently extracts polyphenols, flavonoids, and essential oils, whereas water is widely used in household decoctions and medicinal teas. Hexane, although effective for lipophilic compounds, is unsuitable for food‐grade applications [12, 13]. Consequently, aqueous, ethanolic, and hydroethanolic solvents represent scientifically sound, affordable, and locally relevant extraction strategies [2, 14]. Yet, the influence of solvent polarity on the phytochemical richness and multifunctional bioactivity of Cameroonian spices remains poorly documented.

Cardiometabolic diseases, including obesity, Type 2 diabetes, dyslipidemia, and cardiovascular disorders, are among the leading causes of global morbidity and mortality, largely driven by sedentary lifestyles and poor dietary habits [15, 16]. Their pathogenesis involves insulin resistance, dyslipidemia, impaired glucose homeostasis, and chronic low‐grade inflammation, which collectively promote endothelial dysfunction, oxidative stress, and vascular injury. Dysregulated metabolic regulators such as GLP‐1, irisin, and PGC‐1*α* contribute to obesity‐associated metabolic impairment [16], whereas nitric oxide–dependent dysfunction exacerbates tissue damage in diabetes [17]. Impaired oxygen utilization and vascular inflammation further accelerate cardiovascular disease progression [18]. Although pharmacotherapy and lifestyle modification remain central to management, they face limitations: drugs often target single pathways and may induce adverse effects, whereas lifestyle interventions are difficult to sustain in resource‐constrained settings [19–22]. These shortcomings highlight the need for complementary, multitarget, food‐based strategies.

Bioactive compounds from culinary spices represent promising biochemical modulators of metabolic dysfunction. Their phytochemicals interact with key metabolic checkpoints, including enzymes involved in carbohydrate digestion such as *α*‐amylase and *α*‐glucosidase, whose inhibition can attenuate postprandial increases in blood glucose; lactose metabolism and microbiota‐related carbohydrate processing through *β*‐galactosidase, which contributes to intestinal glycan turnover and influences gut‐derived inflammatory signals; lipid absorption via pancreatic lipase, a major determinant of dietary fat uptake; cholesterol biosynthesis through HMG‐CoA reductase, a central regulator of endogenous cholesterol production; and vascular regulation mediated by ACE, which modulates blood pressure and endothelial function. Together, these enzymes represent interconnected biochemical pathways involved in glucose homeostasis, lipid handling, cholesterol metabolism, gut‐mediated inflammation, and vascular tone, key processes underlying cardiometabolic disorders [23, 24]. They also inhibit inflammatory mediators such as cyclooxygenases (cyclooxygenase‐1 [COX‐1] and cyclooxygenase‐2 [COX‐2]) and 5‐lipoxygenase (5‐LOX), which drive prostaglandin and leukotriene biosynthesis [25, 26]. By simultaneously attenuating oxidative stress, metabolic dysregulation, and inflammation, spice‐derived bioactives offer culturally relevant, nutritionally sustainable strategies for cardiometabolic disease prevention [27].

Among Cameroonian spices, *M. whitei* roots, *S. zenkeri* stem bark, *S. aromaticum* flower buds, and *X. aethiopica* fruits are widely used for their therapeutic properties [3, 4, 23, 28]. *M. whitei* is traditionally valued for its fortifying effects against fatigue and stress [29, 30], whereas *S. aromaticum* is renowned for its digestive, anti‐inflammatory, antimicrobial, and analgesic activities [31, 32]. A review on *M. whitei* highlights its rich profile of phenolic acids, flavonoids, and terpenoids and emphasizes the strong influence of solvent polarity on the recovery of these constituents, particularly in relation to antioxidant and metabolic activities [33]. Similarly, a review on *S. aromaticum* details its high eugenol content and abundant flavonoids and tannins and reports robust antioxidant and biological properties that vary substantially with extraction solvent [34]. *S. zenkeri* is traditionally used to relieve infections and muscular pain [35], whereas *X. aethiopica* is appreciated for its digestive, anti‐inflammatory, antimicrobial, analgesic, postpartum tonic, and immune‐boosting effects [36, 37]. These species are particularly rich in polyphenols, flavonoids, and tannins [37], phytochemicals widely associated with antioxidant and enzyme‐modulatory effects, although such associations remain indicative at this stage and do not imply direct mechanistic attribution. Taken together, their cultural prominence, diverse traditional indications, contrasting botanical origins, and the distinct plant parts traditionally employed make these four spices particularly relevant for exploring how solvent polarity shapes extract composition and multifunctional bioactivity.

Previous investigations, including the work of Halle et al. [23], have shown that Cameroonian spices possess enzyme‐modulatory properties relevant to metabolic regulation. Building on these initial observations, the present study focuses on a broader panel of cardiometabolic targets that reflect both the traditional uses and the phytochemical composition of these spices. Within this biochemical context, in vitro enzyme‐based and antioxidant assays remain widely used as preliminary tools to explore the mechanistic potential of plant extracts, offering a coherent framework for characterizing enzyme‐modulatory and antioxidant properties and for examining how extraction parameters, such as solvent polarity, may influence the activity profiles of spice‐derived phytochemicals. By quantifying activities across carbohydrate digestion, lipid absorption, cholesterol biosynthesis, vascular regulation, and inflammatory signaling, together with complementary antioxidant measurements, the study is aimed at generating an integrated profile of their multifunctional bioactivity. This approach directly supports the objective of determining how solvent polarity influences extract composition and biological performance while providing a comprehensive biochemical characterization relevant to cardiometabolic health.

This study examines how solvent type influences extraction efficiency, phytochemical composition, and biological activity of *M. whitei*, *S. zenkeri*, *S. aromaticum*, and *X. aethiopica*, with emphasis on their relevance to cardiometabolic health. By comparing aqueous, ethanolic, and hydroethanolic extracts, it evaluates how solvent polarity shapes the recovery of key phenolic constituents and the ability of the extracts to modulate major digestive and metabolic enzymes, alongside antioxidant and anti‐inflammatory responses. As an exploratory investigation, it delivers the first comprehensive multisolvent assessment of these four Cameroonian spices across multiple antioxidant assays and nine metabolic and inflammatory enzyme targets, revealing previously unreported functional activities and clarifying how extraction conditions influence biochemical potency. Such insights are essential for guiding future characterization of extract composition, optimizing extraction strategies, and informing the development of culturally relevant nutraceuticals and functional foods.

## 2. Materials and Methods

### 2.1. Plant Collection and Extraction

This study investigated four Cameroonian spices traditionally used for culinary and medicinal purposes: the roots of *M. whitei* (Hook. f.) Skeels, the bark of *S. zenkeri* Harms, the flower buds of *S. aromaticum*, and the seeds of *X. aethiopica* (Dunal) A. Rich. Dried plant materials were purchased from the Gare Market in Douala, Cameroon. Species identity was first confirmed by a botany lecturer in the Department of Plant Biology, University of Douala, and subsequently authenticated at the National Herbarium of Cameroon (Yaoundé), where voucher specimens were deposited under the following reference numbers: *M. whitei* (No. 34180 SRFCam), *S. zenkeri* (No. 44803 SRFCam), *S. aromaticum* (No. 66995/HNC), and *X. aethiopica* (No. 1719/SRF/Cam).

Each sample was cleaned, air‐dried under shade, and ground into fine powder using a mechanical grinder (SOKANY, RoHS‐certified, China). The powdered materials were stored in sterile, airtight containers at room temperature (27^°^C ± 2^°^C) until extraction.

Extraction was performed using three solvents, aqueous, ethanolic, and hydroethanolic (30:70 *v*/*v*), following the protocol of Morakinyo et al. [38] with minor modifications. The 30:70 hydroethanolic ratio was selected because this polarity balance is widely reported to enhance the solubilization of semipolar phenolics and flavonoids, offering a compromise between the extractive strengths of water and ethanol. For each extraction, 30 g of plant powder was placed in an Erlenmeyer flask and homogenized with 300 mL of the respective solvent: distilled water (aqueous), ethanol (ethanolic), or a hydroethanolic mixture prepared from 210 mL ethanol and 90 mL distilled water. All mixtures were macerated for 72 h at room temperature (27^°^C ± 2^°^C) with continuous stirring.

After maceration, extracts were sequentially filtered through muslin cloth and Whatman No. 4 filter paper. Filtrates were concentrated in a drying oven (Binder FD 53, Binder GmbH, Tuttlingen, Germany) at 55°C for approximately 3 days to obtain dry extracts. The dried extracts were stored in sterile, airtight containers at 4°C until analysis. Extraction yield was calculated as the ratio of the mass of dry extract obtained to the initial mass of plant powder.

### 2.2. Enzymes

This study employed a comprehensive panel of enzymes selected for their central roles in the pathophysiology of metabolic syndrome and associated inflammatory processes. All enzymes and reagents were obtained from Sigma‐Aldrich (St. Louis, MO, United States) and handled according to the manufacturer′s specifications to ensure biological integrity.

Six metabolic enzymes were used: *α*‐amylase (EC 3.2.1.1; porcine pancreas, catalog A4268), *α*‐glucosidase (EC 3.2.1.20; *Bacillus stearothermophilus*, catalog G3651), *β*‐galactosidase (EC 3.2.1.23; *Escherichia coli*, catalog G5635), pancreatic lipase (EC 3.1.1.3; porcine pancreas, catalog L0382), HMG‐CoA reductase (EC 1.1.1.34; recombinant human catalytic domain, catalog H7039), and ACE (EC 3.4.15.1; rabbit lung, catalog A6778). These enzymes regulate carbohydrate and lipid digestion, cholesterol biosynthesis, and vascular tone, making them critical therapeutic targets in obesity, diabetes, dyslipidemia, and hypertension.

To complement this metabolic panel, three enzymes involved in inflammatory signaling were also assessed: COX‐1 (EC 1.14.99.1; sheep, catalog C0733), COX‐2 (EC 1.14.99.1; human recombinant expressed in insect cells, catalog C0858), and 5‐LOX (EC 1.13.11.34; human recombinant expressed in *Spodoptera frugiperda*, catalog 437996). These enzymes catalyze the biosynthesis of prostaglandins and leukotrienes, potent mediators of vascular inflammation and atherosclerosis, thereby providing an integrated view of spice‐derived bioactivity across metabolic and inflammatory pathways.

To ensure assay validity, positive reference inhibitors previously reported in related studies were included for each enzymatic target at fixed concentrations prior to testing the plant extracts. Specifically, acarbose was used for *α*‐amylase and *α*‐glucosidase, D‐galactonolactone for *β*‐galactosidase, orlistat for pancreatic lipase, pravastatin for HMG‐CoA reductase, captopril for ACE, and indomethacin for COX‐1/COX‐2 and 5‐LOX. These controls confirmed that the assay systems responded as expected and provided internal benchmarks for comparison, ensuring reproducibility and reliability of the experimental outcomes.

### 2.3. Preparation of Extract Solutions for Analysis

For all analyses, crude extract solutions were prepared at a final concentration of 1 mg/mL using distilled water or the appropriate assay buffer. To ensure complete solubilization, each dried extract was first dispersed in a minimal volume of dimethyl sulfoxide (DMSO) and subsequently diluted to the required volume with the selected solvent. The final DMSO concentration in all assay wells was maintained below 0.5% (*v*/*v*), a level reported to have negligible effects on enzymatic and antioxidant readouts [39, 40].

Matched solvent controls containing the same DMSO concentration but no extract were included in every experiment to correct for solvent‐related interference. In addition, an extract‐specific blank was prepared for each extract and concentration to account for intrinsic color or turbidity. This blank contained the extract, buffer, and DMSO, but no enzyme, substrate, or radical was read at the same wavelength as the corresponding reaction mixture, and its absorbance was subtracted from the sample readings. This correction was systematically applied to all antioxidant and enzyme inhibition assays.

All subsequent assays were performed using three independent biological replicates, each measured with three technical readings to ensure instrumental precision.

### 2.4. Phytochemical Quantification

The phenolic composition of the spice extracts was quantified to assess their chemical richness and bioactive potential. Three parameters were evaluated: total polyphenol contents (TPCs), flavonoids, and condensed tannins. Extracts were prepared in 0.5% DMSO and subsequently diluted in distilled water, and all assays were performed using a UV–Vis spectrophotometer (Shimadzu UV‐1800, Japan). For each assay, a reagent blank consisting of distilled water with 0.5% (*v*/*v*) DMSO but without extract or standard was used to correct background absorbance.

TPC was determined using the Folin–Ciocalteu method [41]. In brief, 30 *μ*L of extract was mixed with 2.5 mL of Folin–Ciocalteu reagent (diluted 1:10) and incubated for 2 min in the dark at room temperature (27^°^C ± 2^°^C). Subsequently, 2 mL of sodium carbonate solution (75 g/L) was added, and after 15 min, absorbance was measured at 760 nm. Gallic acid (0–200 mg/L) served as the calibration standard, and results were expressed as milligrams of gallic acid equivalents per gram of extract.

Flavonoid content was quantified using the aluminum chloride colorimetric method [42, 43]. A mixture of 2.5 mL extract, 0.75 mL of 5% sodium nitrite, and 0.75 mL of 10% aluminum chloride was incubated for 5 min, followed by the addition of 5 mL of 1 M sodium hydroxide. Absorbance was recorded at 510 nm. Quercetin (10–100 mg/L) was used as the reference standard, and results were expressed as milligrams of quercetin equivalents per gram of extract.

Condensed tannins were measured using the vanillin–HCl method [44, 45]. For each sample, 50 *μ*L of extract were combined with 1.5 mL of 4% vanillin in methanol and 750 *μ*L of concentrated hydrochloric acid. After vigorous mixing and a 20‐min reaction at room temperature (27^°^C ± 2^°^C), absorbance was measured at 550 nm. Tannic acid (10–100 mg/L) served as the calibration standard, and results were expressed as milligrams of tannic acid equivalents per gram of extract.

### 2.5. Enzyme Inhibition Assays

The inhibitory potential of the spice extracts against key metabolic enzymes was assessed using microplate‐based spectrophotometric assays, following manufacturers′ guidelines and the protocol of Halle et al. [23] with minor modifications. Test samples and reference inhibitors were prepared across a concentration range of 1–2000 *μ*g/mL in the appropriate buffer: phosphate buffer (100 mM, pH 6.9) for *α*‐amylase, *α*‐glucosidase, *β*‐galactosidase, pancreatic lipase, and HMG‐CoA reductase and Tris–HCl buffer (100 mM, pH 8.0) for ACE. Percentage inhibition was calculated relative to untreated controls, and IC_50_ values were obtained from nonlinear regression using a four‐parameter logistic model. For each assay, a reagent blank containing the substrate in buffer with 0.5% (*v*/*v*) DMSO or reference inhibitor but without enzyme was used to correct background absorbance. Absorbance was recorded using a BioTek Synergy HTX Multi‐Mode Microplate Reader (Agilent Technologies, United States).

The *α*‐amylase assay was based on the reaction of reducing sugars with 3,5‐dinitrosalicylic acid (DNS), producing a red–orange complex measurable at 540 nm [46, 47]. Reaction mixtures (200 *μ*L) contained 100 *μ*L soluble starch (1% *w*/*v*), 50 *μ*L *α*‐amylase (1 U/mL), and 50 *μ*L extract or acarbose. After incubation at 37°C for 30 min, the reaction was stopped with 100 *μ*L DNS reagent, heated at 100°C for 5 min, cooled, and read at 540 nm.

For *α*‐glucosidase, inhibition was determined using p‐nitrophenyl‐*α*‐D‐glucopyranoside (pNPG) as substrate, releasing p‐nitrophenol detectable at 405 nm [48, 49]. Each 200 *μ*L reaction contained 100 *μ*L pNPG (2 mM), 50 *μ*L *α*‐glucosidase (1 U/mL), and 50 *μ*L extract or acarbose. After incubation at 37°C for 30 min, the reaction was terminated with 50 *μ*L sodium carbonate (0.1 M), and absorbance was measured at 405 nm.

*β*‐Galactosidase inhibition was assessed using o‐nitrophenyl‐*β*‐D‐galactopyranoside (oNPG), which releases o‐nitrophenol detectable at 420 nm [50, 51]. Reaction mixtures (200 *μ*L) consisted of 100 *μ*L oNPG (2 mM), 50 *μ*L *β*‐galactosidase (1 U/mL), and 50 *μ*L extract or D‐galactonolactone. After incubation at 37°C for 30 min, the reaction was stopped with 50 *μ*L sodium carbonate (1 M), and absorbance was recorded at 420 nm.

Pancreatic lipase inhibition was evaluated using p‐nitrophenyl palmitate (pNPP) as substrate, with p‐nitrophenol release monitored at 405 nm [52, 53]. pNPP was dissolved in isopropanol and emulsified in phosphate buffer (pH 7.4) containing 0.5% Triton X‐100. Reaction mixtures (200 *μ*L) contained 100 *μ*L pNPP emulsion (0.5 mM), 50 *μ*L pancreatic lipase (1 mg/mL), and 50 *μ*L extract or orlistat. After incubation at 37°C for 30 min, the reaction was stopped with 50 *μ*L sodium carbonate (0.1 M). To exclude nonspecific inhibition due to aggregation or turbidity, parallel assays were performed with 0.01% Triton X‐100, and extract solutions were visually inspected to confirm the absence of precipitation. Absorbance was measured at 405 nm.

HMG‐CoA reductase inhibition was assessed by monitoring NADPH oxidation during the conversion of HMG‐CoA to mevalonate, measured as a decrease in absorbance at 340 nm [54, 55]. Reaction mixtures (200 *μ*L) contained 100 *μ*L HMG‐CoA (0.1 mM in potassium phosphate buffer, pH 6.9, supplemented with 5 mM MgCl_2_, 1 mM DTT, 50 mM KCl, and 1 mM EDTA), 50 *μ*L NADPH (0.2 mM), and 50 *μ*L HMG‐CoA reductase (0.1 U/mL) with extract or pravastatin. After incubation at 37°C for 30 min, reactions were stopped on ice and read at 340 nm.

ACE inhibitory activity was evaluated using furylacryloyl–phenylalanyl–glycyl–glycine (FAPGG) as a substrate, following established spectrophotometric procedures [56, 57]. Hydrolysis of FAPGG was monitored by the decrease in absorbance at 340 nm. Each 200 *μ*L reaction contained 100 *μ*L FAPGG (5 mM in Tris–HCl buffer, pH 8.0, with 300 mM NaCl), 50 *μ*L ACE (0.1 U/mL), and 50 *μ*L extract or captopril. After incubation at 37°C for 30 min, reactions were stopped on ice, and absorbance was recorded against the extract‐specific blank.

### 2.6. Antioxidant Activity Evaluation

The antioxidant potential of the spice extracts was assessed using three complementary in vitro spectrophotometric assays: DPPH radical scavenging, ABTS radical cation decolorization, and ferric reducing antioxidant power (FRAP). Extracts were prepared in distilled water, and both samples and vitamin C (reference standard) were tested across concentrations ranging from 1 to 2000 *μ*g/mL. All assays were performed in 96‐well microplates, and absorbance was recorded using a microplate spectrophotometer equipped with pathlength correction to normalize readings to a 1 cm optical path. IC_50_ values were calculated for DPPH and ABTS assays, while EC_50_ values (absorbance of 0.5) were determined for FRAP. These parameters were obtained from dose–response curves plotting scavenging activity or absorbance against concentration. For each assay, a reagent blank containing the respective radical or FRAP reagent mixed with distilled water and 0.5% (*v*/*v*) DMSO but without extract or standard was used to correct background absorbance.

The DPPH assay followed the methods of Sigma‐Aldrich [58] and Blois [59]. Antioxidant activity was quantified by the reduction of the purple DPPH radical to a yellow form. For each test, 500 *μ*L of extract was mixed with 1000 *μ*L of DPPH solution (0.024 g in 300 mL of methanol), incubated in the dark at room temperature (27^°^C ± 2^°^C) for 20 min, and absorbance was measured at 517 nm.

The ABTS assay was performed according to Brand‐Williams et al. [60]. The ABTS radical cation (ABTS•^+^) was generated by reacting 7.4 mM ABTS in ethanol with 2.6 mM potassium persulfate in water for 24 h in the dark. For each reaction, 500 *μ*L of extract was added to 1000 *μ*L of ABTS•^+^ solution, incubated for 20 min at room temperature (27^°^C ± 2^°^C) in the dark, and absorbance was recorded at 734 nm.

The FRAP assay followed the procedure of Ilyasov et al. [61]. Antioxidant capacity was assessed by the reduction of Fe^3+^ to Fe^2+^ in the presence of 2,4,6‐tripyridyl‐s‐triazine (TPTZ), forming a blue Fe^2+^–TPTZ complex measurable at 593 nm. The FRAP reagent was freshly prepared by mixing acetate buffer (300 mM, pH 3.6), TPTZ solution (10 mM in 40 mM HCl), and FeCl_3_·6H_2_O solution (20 mM) in a 10:1:1 ratio. For each reaction, 100 *μ*L of extract was added to 3 mL of FRAP reagent, incubated at 37°C for 30 min, and absorbance was measured at 593 nm.

### 2.7. Anti‐Inflammatory Activity Evaluation

The anti‐inflammatory potential of the spice extracts was assessed by evaluating their ability to inhibit COX‐1, COX‐2, and 5‐LOX, key enzymes involved in eicosanoid biosynthesis. Extracts were prepared in the appropriate buffer for each assay: Tris–HCl buffer (100 mM, pH 8.0) for COX‐1 and COX‐2 and borate buffer (50 mM, pH 9.0) for 5‐LOX. Indomethacin served as the reference inhibitor. Both extracts and the reference inhibitor were tested at concentrations ranging from 1 to 2000 *μ*g/mL. Inhibition was expressed relative to untreated controls, and IC_50_ values were obtained from nonlinear regression of dose–response curves. For each assay, a reagent blank containing the substrate in buffer with 0.5% (*v*/*v*) DMSO or the reference inhibitor but without enzyme was used to correct background absorbance, and all experiments were performed using the same microplate reader described above.

COX inhibition assays followed the spectrophotometric procedures of Alkhatib et al. [25], Sud′ina et al. [26], and Benzie and Devaki and Sigma‐Aldrich [62, 63]. Reaction mixtures (1 mL) were prepared in Tris–HCl buffer containing 5 mM EDTA, 0.5 mM phenol, and purified COX‐1 or COX‐2 enzyme. Hematin was used as an activator. Diluted extracts were added prior to a 10‐min preincubation at 37°C. Reactions were initiated by adding arachidonic acid to a final concentration of 100 *μ*M (10 *μ*L of a 10 mM stock). After 2–5 min, prostaglandin formation was quantified at 590 nm. Reactions were stopped by immediate cooling on ice, and absorbance was measured against the blank.

The 5‐LOX inhibition assay was performed according to Alkhatib et al. [25]. Reaction mixtures (1 mL) were prepared in borate buffer, and diluted extracts were added at the desired concentrations. After a 5‐min preincubation at 25°C, reactions were initiated by adding arachidonic acid to a final concentration of 20 *μ*M (2 *μ*L of a 10 mM stock). Formation of conjugated dienes, indicative of 5‐LOX activity, was monitored at 234 nm over 5 min. Reactions were stopped by cooling on ice, and absorbance was recorded against the blank.

### 2.8. Statistical Analysis

All experimental data were first compiled in Microsoft Excel (Microsoft Office, United States) prior to statistical processing. Statistical analyses were performed using GraphPad Prism Version 8.4 (GraphPad Software, United States). A two‐way analysis of variance (ANOVA) was applied to assess the effects of plant species and extraction solvent on each biochemical and biological parameter. When significant differences were detected (*p* < 0.05), Tukey′s post hoc test was used for pairwise comparisons within each factor. Quantitative results were expressed as mean ± standard deviation (SD), and statistical significance was set at *p* < 0.05 for all analyses.

Pearson′s correlation analysis was additionally conducted to examine the relationships between phytochemical constituents (TPCs, flavonoids, and tannins) and the antioxidant, metabolic, and inflammatory activities measured across the spice extracts. Correlation coefficients (*r*) were calculated for each pair of variables, with significance set at *p* < 0.05. The resulting correlation matrix was visualized as a heat map to facilitate interpretation of the strength and direction of associations among biochemical parameters and biological endpoints.

To enable a coherent visual comparison across all biochemical and biological parameters, raw data were normalized using the min–max method, according to the formula *X*norm = (*X* − *X*min)/(*X*max − *X*min). This normalization was applied separately for each parameter using all 12 values (four spices × three solvents). The normalized data were then grouped by solvent and displayed as boxplots, allowing the solvent effect to be illustrated independently of the original units and the initial amplitude of variation.

## 3. Results and Discussion

### 3.1. Extraction Yield

**Figure 1:**
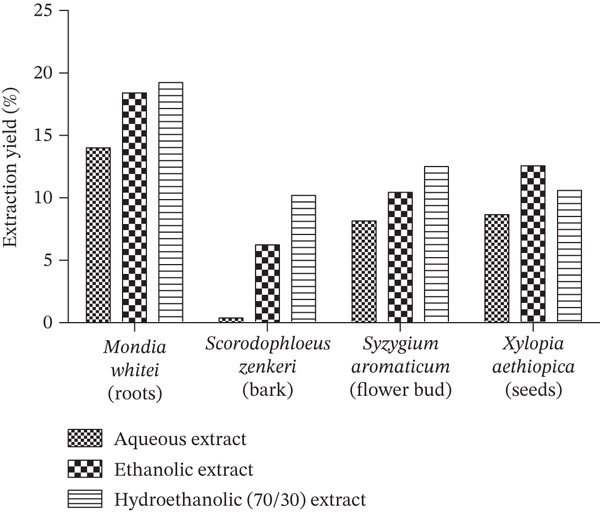
Comparative extraction yields of the three spices under different solvent conditions (aqueous, ethanolic, and hydroethanolic [30/70]).

Extraction yields varied substantially across species and solvent systems, confirming the strong influence of solvent polarity on the recovery of extractable matter (Figure 1). *M. whitei* produced the highest yields, reaching 19% with hydroethanol, consistent with its abundance of polar and semipolar constituents and in agreement with previous reports highlighting the efficiency of ethanol–water mixtures for this species [64].

*X. aethiopica* showed intermediate yields (8%–13%), with ethanol outperforming water, reflecting its high content of essential oils and semipolar flavonoids that exhibit limited aqueous solubility. Similar solvent‐dependent patterns have been described for lipophilic spice matrices [10, 65].

Aqueous extraction of *S. zenkeri* yielded only 0.4%, likely due to its dense, lignified bark and low levels of water‐soluble metabolites. Yield increased markedly with hydroethanol (10%), illustrating the ability of mixed solvents to disrupt rigid tissues and enhance diffusion of semipolar compounds, as previously noted for bark‐derived materials [37, 65].

*S. aromaticum* followed a comparable trend, with yields of 8% (aqueous), 10% (ethanolic), and 13% (hydroethanolic), consistent with its high content of semipolar phenolics that are more efficiently solubilized in ethanol‐rich systems.

Overall, hydroethanolic extraction improved yields for three of the four species, whereas ethanolic extraction was more effective for *X. aethiopica*, indicating that solvent polarity influences extraction efficiency in a species‐dependent manner rather than uniformly favoring hydroethanol.

### 3.2. Quantitative Phytochemical Screening

The concentrations of TPCs, flavonoids, and condensed tannins varied significantly across species and solvents (Table 1). For TPCs, *S. zenkeri* showed the highest values across solvents; for example, its ethanolic extract reached 595 mg GAE/g, consistent with previous observations on the efficiency of organic solvents for phenolic recovery [10]. In *M. whitei*, hydroethanolic and aqueous extracts displayed comparable polyphenol levels, whereas the ethanolic extract was significantly lower. *S. aromaticum* showed intermediate values with clear solvent‐dependent differences, in line with the known solubility patterns of its eugenol‐rich phenolics [65]. *X. aethiopica* presented the lowest polyphenol content, with hydroethanolic extract significantly exceeding its aqueous and ethanolic forms, consistent with earlier reports of its moderate phenolic density [66].

**Table 1:** Content of total polyphenols, flavonoids, and condensed tannins in spice extracts by solvent type.

Compound	Extraction type	*Mondia whitei* (roots)	*Scorodophloeus zenkeri* (bark)	*Syzygium aromaticum* (flower bud)	*Xylopia aethiopica* (seeds)
Total polyphenols (mg GAE/g)	Aqueous	470 ± 4ᵇᵝ	579 ± 2ᵈᵅ	298 ± 3ᵇᵅ	256 ± 5ᵃᵅ
	Ethanolic	372 ± 2ᶜᵅ	595 ± 5ᵈᵞ	326 ± 9ᵇᵝ	268 ± 9ᵃᵝ
	Hydroethanolic (30/70)	482 ± 2ᵇᵞ	590 ± 6ᵈᵝ	359 ± 5ᵇᵞ	303 ± 3ᵃᵞ
Flavonoids (mg QE/g)	Aqueous	22 ± 0.1ᶜᵝ	19 ± 0.1ᵃᵅ	24 ± 1ᵈᵅ	20 ± 0.1ᵇᵅ
	Ethanolic	18 ± 0.1ᵃᵅ	18 ± 0.1ᵃᵅ	29 ± 0.2ᵈᵞ	19 ± 0.2ᵃᵝ
	Hydroethanolic (30/70)	21 ± 0.1ᵇᵞ	20 ± 0.0ᵃᵝ	30 ± 0.1ᵈᵞ	20 ± 0.0ᵃᵅ
Condensed tannins (mg TAE/g)	Aqueous	43 ± 1ᶜᵞ	41 ± 2ᵇᵞ	38 ± 0.3ᵃᵅ	37 ± 0.5ᵃᵞ
	Ethanolic	39 ± 1ᶜᵅ	37 ± 2ᵇᵅ	37 ± 0.2ᵇᵅ	35 ± 0.4ᵃᵅ
	Hydroethanolic (30/70)	40 ± 1ᵇᵝ	40 ± 1ᶜᵝ	39 ± 1ᵇᵅ	36 ± 0.2ᵃᵝ

*Note:* Values are expressed as mean ± SD (*n* = 3). A two‐way ANOVA (factors: species × solvent) followed by Tukey′s post hoc test was performed. For each solvent, different lowercase superscript letters (a–d) indicate significant differences among species (*p* < 0.05). For each species, different Greek superscript letters (*α*–*γ*) indicate significant differences among solvents (*p* < 0.05). Lowercase and Greek superscripts represent independent comparison sets and should not be compared across rows or columns.

Abbreviations: GAE, gallic acid equivalent; QE, quercetin equivalent; TAE, tannic acid equivalent.

Flavonoid levels also exhibited species‐specific solvent effects. In *M. whitei*, aqueous and hydroethanolic extracts contained significantly more flavonoids than the ethanolic extract, suggesting a predominance of water‐soluble flavonoid glycosides. *S. zenkeri* showed no significant variation across solvents. *S. aromaticum* had the highest flavonoid concentrations overall, with hydroethanolic extract reaching 30 mg QE/g, consistent with its documented richness in flavonoid aglycones [67]. In *X. aethiopica*, aqueous and hydroethanolic extracts did not differ significantly, whereas the ethanolic extract showed a lower value, in agreement with recent findings [66].

Condensed tannins followed similar patterns. In *M. whitei*, aqueous extract contained significantly more tannins than the ethanolic extract. *S. zenkeri* showed comparable tannin levels in aqueous and hydroethanolic extracts, both significantly higher than ethanol. *S. aromaticum* exhibited moderate tannin content with significant solvent‐dependent differences, reflecting the affinity of its hydrolyzable tannins for polar media [67, 68]. In *X. aethiopica*, aqueous and hydroethanolic extracts were statistically similar, while the ethanolic extract was significantly lower.

Overall, the updated statistical analysis confirms that solvent effects must be interpreted within each species and only when supported by significant differences. The revised legend and simplified superscripts ensure clearer and more accurate interpretation of phytochemical variation.

### 3.3. Enzyme Inhibitory Activity

The inhibitory activity of *M. whitei*, *S. zenkeri*, *S. aromaticum*, and *X. aethiopica* was evaluated against six metabolic enzymes relevant to cardiometabolic regulation (Table 2). The extracts displayed distinct species‐ and solvent‐dependent profiles, reflecting differences in extract composition and polarity. As these assays are cell‐free biochemical systems, the results provide preliminary indications of inhibitory potential and should be interpreted within the limits of in vitro screening.

**Table 2:** IC_50_ values (micrograms per milliliter) of spice extracts for enzyme inhibition.

Enzyme target	Extraction type	*Mondia whitei* (roots)	*Scorodophloeus zenkeri* (bark)	*Syzygium aromaticum* (flower bud)	*Xylopia aethiopica* (seeds)	Reference inhibitor ^∗^
*α*‐Amylase	Aqueous	0.2 ± 0.0ᵇᵝ	424 ± 22ᶜᵅ	0.1 ± 0.0ᵃᵅ	0.2 ± 0.0ᵇᵅ	57 ± 3
	Ethanolic	0.01 ± 0.0ᵃᵅ	712 ± 29ᵈᵞ	0.1 ± 0.0ᵇᵅ	34 ± 0.3ᶜᵞ	
	Hydroethanolic (30/70)	5 ± 0.2ᶜᵞ	438 ± 7ᵈᵝ	0.1 ± 0.0ᵃᵅ	2 ± 1ᵇᵝ	
*α*‐Glucosidase	Aqueous	59 ± 4ᵇᵝ	50 ± 4ᵃᵝ	80 ± 2ᵈᵞ	72 ± 5ᶜᵞ	151 ± 9
	Ethanolic	51 ± 4ᵇᵅ	44 ± 3ᵃᵅ	62 ± 4ᶜᵅ	65 ± 5ᵈᵅ	
	Hydroethanolic (30/70)	189 ± 8ᶜᵞ	198 ± 7ᵈᵞ	72 ± 4ᵃᵝ	83 ± 5ᵇᵝ	
*β*‐Galactosidase	Aqueous	1150 ± 40ᵇᵝ	1300 ± 30ᵈᵞ	1030 ± 20ᵃᵅ	1290 ± 30ᶜᵞ	590 ± 10
	Ethanolic	1220 ± 20ᶜᵞ	1040 ± 40ᵃᵅ	1190 ± 30ᵇᵞ	1340 ± 30ᵈᵞ	
	Hydroethanolic (30/70)	1080 ± 30ᵃᵅ	1200 ± 80ᵇᵝ	1120 ± 30ᵃᵝ	1260 ± 10ᶜᵝ	
Lipase	Aqueous	720 ± 38ᶜᵝ	10 ± 2ᵃᵅ	12 ± 1ᵃᵅ	486 ± 46ᵇᵞ	281 ± 15
	Ethanolic	649 ± 24ᵈᵅ	153 ± 6ᵇᵝ	18 ± 1ᵃᵝ	390 ± 44ᶜᵝ	
	Hydroethanolic (30/70)	1110 ± 10ᵈᵞ	494 ± 20ᶜᵞ	22 ± 1ᵃᵞ	316 ± 25ᵇᵅ	
HMG‐CoA reductase	Aqueous	5 ± 0.4ᵇᵝ	4 ± 0.3ᵃᵝ	5 ± 0.2ᵇᵝ	7 ± 0.5ᶜᵞ	7 ± 0.8
	Ethanolic	4 ± 0.3ᵃᵅ	3 ± 0.3ᵃᵅ	4 ± 0.2ᵇᵅ	6 ± 0.4ᶜᵝ	
	Hydroethanolic (30/70)	3 ± 0.3ᵃᵅ	4 ± 0.4ᵇᵝ	5 ± 0.3ᶜᵝ	4 ± 0.3ᵇᵅ	
ACE	Aqueous	12 ± 0.8ᶜᵝ	11 ± 0.7ᵇᵞ	6 ± 0.2ᵃᵅ	18 ± 1ᵈᵝ	14 ± 0.4
	Ethanolic	10 ± 0.7ᵇᵅ	9 ± 0.6ᵇᵅ	6 ± 0.2ᵃᵅ	15 ± 1ᶜᵅ	
	Hydroethanolic (30/70)	10 ± 0.3ᵇᵅ	10 ± 0.5ᵇᵝ	6 ± 0.2ᵃᵅ	15 ± 0.7ᶜᵅ	

*Note:* Values are expressed as mean ± SD (*n* = 3). A two‐way ANOVA (factors: species × solvent) followed by Tukey′s post hoc test was performed. For each solvent, different lowercase superscript letters (a–d) indicate significant differences among species (*p* < 0.05). For each species, different Greek superscript letters (*α*–*γ*) indicate significant differences among solvents (*p* < 0.05). Lowercase and Greek superscripts represent independent comparison sets and should not be compared across rows or columns.

Abbreviations: ACE, angiotensin‐converting enzyme; HMG‐CoA reductase, *β*‐hydroxy *β*‐methylglutaryl‐CoA reductase; IC_50_, half‐maximal inhibitory.

^∗^Reference inhibitors: acarbose for α‐amylase and α‐glucosidase, D‐galactonolactone for β‐galactosidase, orlistat for lipase, pravastatin for HMG‐CoA reductase, and captopril for ACE.

In the *α*‐amylase assay, several extracts exhibited very low IC_50_ values, including ethanolic *M. whitei* (0.01 *μ*g/mL), aqueous *X. aethiopica* (0.2 *μ*g/mL), and aqueous or hydroethanolic *S. aromaticum* (0.1 *μ*g/mL), all markedly lower than acarbose (57 *μ*g/mL). Such values likely reflect strong interactions under assay conditions and may also be influenced by matrix effects inherent to crude extracts. *S. zenkeri* showed significantly weaker inhibition across solvents. These patterns are consistent with previous observations describing solvent‐dependent extraction of constituents capable of interacting with *α*‐amylase [69, 70].

For *α*‐glucosidase, most extracts displayed IC_50_ values below acarbose (151 *μ*g/mL). Ethanolic extracts of *S. zenkeri*, *M. whitei*, and *X. aethiopica* were among the most active (e.g., 44–65 *μ*g/mL), whereas hydroethanolic extracts of *M. whitei* and *S. zenkeri* showed significantly higher IC_50_ values (around 189–198 *μ*g/mL). These differences highlight the influence of solvent polarity on the extraction of constituents previously reported to modulate *α*‐glucosidase activity in these species [66, 69, 71–73].

*β*‐Galactosidase inhibition was generally weak, with IC_50_ values exceeding that of D‐galactonolactone (590 *μ*g/mL). Slightly lower values were observed in hydroethanolic *M. whitei* (1080 *μ*g/mL), ethanolic *S. zenkeri* (1040 *μ*g/mL), and aqueous *S. aromaticum* (1030 *μ*g/mL). These trends align with previous reports describing limited *β*‐galactosidase inhibition in these species [37, 70], although some studies have suggested that polyphenol‐rich extracts may influence *β*‐galactosidase‐related pathways indirectly [74–76].

Pancreatic lipase inhibition varied significantly across species and solvents. Aqueous *S. zenkeri* (10 *μ*g/mL) and *S. aromaticum* (12–22 *μ*g/mL) showed the lowest IC_50_ values, below orlistat (281 *μ*g/mL) under the same assay conditions. These results indicate strong inhibitory potential in vitro, although crude extracts may influence absorbance‐based detection. *X. aethiopica* showed moderate inhibition (e.g., 316–486 *μ*g/mL), whereas *M. whitei* exhibited significantly weaker activity. The solvent‐dependent differences observed are consistent with previously reported variations in extract composition [66].

All species showed notable inhibition of HMG‐CoA reductase, with several extracts presenting IC_50_ values close to or below pravastatin (7 *μ*g/mL). For example, ethanolic *S. zenkeri* and hydroethanolic *M. whitei* showed values around 3–4 *μ*g/mL. These results indicate strong inhibitory potential under assay conditions, in agreement with earlier reports describing constituents capable of modulating cholesterol biosynthesis in these species [69, 70].

ACE inhibition was strongest in *S. aromaticum* (aqueous and ethanolic, 6 *μ*g/mL), with values lower than captopril (14 *μ*g/mL) under the same conditions. *S. zenkeri* and *M. whitei* also showed strong inhibition (around 9–12 *μ*g/mL), whereas *X. aethiopica* exhibited moderate activity. These results are consistent with previous studies reporting ACE‐modulating compounds in these species [77–79].

Overall, the enzyme inhibition results highlight solvent‐dependent differences in extract performance. The very low IC_50_ values observed for several crude extracts likely reflect strong interactions under assay conditions and may also be influenced by matrix effects or assay sensitivity. These findings provide preliminary biochemical insight into the cardiometabolic relevance of the investigated spices while underscoring the exploratory nature of in vitro assays.

### 3.4. Antioxidant Activity

The antioxidant potential of *S. zenkeri*, *M. whitei*, *S. aromaticum*, and *X. aethiopica* was evaluated using DPPH, ABTS, and FRAP assays (Table 3). The results showed clear interspecies and solvent‐dependent variations, reflecting differences in extract composition and polarity. As these assays rely on absorbance‐based detection, the values obtained represent in vitro reactivity and should be interpreted within the methodological limits of these rapid screening systems.

**Table 3:** IC_50_/EC_50_ values (micrograms per milliliter) for antioxidant activity of spice extracts.

Assay	Extraction type	*Mondia whitei* (roots)	*Scorodophloeus zenkeri* (bark)	*Syzygium aromaticum* (flower bud)	*Xylopia aethiopica* (seeds)	Reference (vitamin C)
DPPH	Aqueous	0.5 ± 0.1ᵃᵝ	0.8 ± 0.2ᵇᵝ	4 ± 0.1ᵈᵞ	1.4 ± 0.4ᶜᵞ	12 ± 1
	Ethanolic	0.1 ± 0.0ᵇᵅ	0.02 ± 0.01ᵃᵅ	0.3 ± 0.1ᶜᵅ	0.6 ± 0.1ᵈᵅ	
	Hydroethanolic (30/70)	1.0 ± 0.1ᵈᵞ	0.9 ± 0.1ᶜᵞ	0.5 ± 0.1ᵇᵝ	0.4 ± 0.1ᵃᵝ	
ABTS	Aqueous	0.2 ± 0.1ᵃᵅ	1.4 ± 0.1ᶜᵝ	19 ± 2ᵈᵞ	0.7 ± 0.2ᵇᵝ	16 ± 0.4
	Ethanolic	17 ± 2ᵈᵞ	6 ± 0.6ᵃᵞ	9 ± 1ᵇᵞ	7 ± 0.2ᵃᵞ	
	Hydroethanolic (30/70)	0.4 ± 0.0ᶜᵝ	0.2 ± 0.0ᵃᵅ	0.3 ± 0.0ᵇᵅ	0.3 ± 0.1ᵇᵅ	
FRAP	Aqueous	684 ± 12ᵈᵅ	29 ± 4ᵃᵅ	48 ± 1ᵇᵅ	416 ± 3ᶜᵅ	18 ± 1
	Ethanolic	767 ± 21ᶜᵝ	655 ± 16ᵇᵝ	302 ± 9ᵃᵝ	1120 ± 5ᵈᵝ	
	Hydroethanolic (30/70)	904 ± 13ᵇᵞ	1310 ± 10ᶜᵞ	411 ± 10ᵃᵞ	1620 ± 20ᵈᵞ	

*Note:* Values are expressed as mean ± SD (*n* = 3). A two‐way ANOVA (factors: species × solvent) followed by Tukey′s post hoc test was performed. For each solvent, different lowercase superscript letters (a–d) indicate significant differences among species (*p* < 0.05). For each species, different Greek superscript letters (*α*–*γ*) indicate significant differences among solvents (*p* < 0.05). Lowercase and Greek superscripts represent independent comparison sets and should not be compared across rows or columns.

Abbreviations: ABTS, 2,2 ^′^‐azino‐bis(3‐ethylbenzothiazoline‐6‐sulfonic acid); DPPH, 2,2‐diphenyl‐1‐picrylhydrazyl; EC_50_, effective concentration; FRAP, ferric reducing antioxidant power; IC_50_, half‐maximal inhibitory.

In the DPPH assay, all extracts exhibited lower IC_50_ values than vitamin C (12 *μ*g/mL) under the present conditions. *S. zenkeri* showed the strongest effect, particularly in its ethanolic extract (0.02 *μ*g/mL), consistent with earlier reports describing the high phenolic density of this species [80]. *M. whitei* also demonstrated strong scavenging activity, with the ethanolic extract reaching 0.1 *μ*g/mL, in line with its documented richness in flavonoids and polyphenols [69]. *X. aethiopica* showed moderate but consistent activity, with the hydroethanolic extract performing best (0.4 *μ*g/mL), a trend previously associated with the presence of alkaloids, terpenoids, and phenolic compounds [66]. *S. aromaticum* displayed strong scavenging activity as well, particularly in ethanolic and hydroethanolic extracts (0.3–0.5 *μ*g/mL), coherent with the antioxidant properties of eugenol, gallic acid derivatives, and flavonoid aglycones. These results highlight the influence of solvent polarity on the extraction of constituents capable of donating hydrogen atoms or stabilizing radicals.

The ABTS assay further confirmed the antioxidant capacity of the extracts, with several samples showing IC_50_ values below vitamin C (16 *μ*g/mL). *X. aethiopica* was most active in its hydroethanolic extract (0.3 *μ*g/mL), illustrating the ability of mixed solvents to recover both hydrophilic and lipophilic constituents. *M. whitei* showed peak inhibition in the aqueous extract (0.2 *μ*g/mL), consistent with its high levels of water‐soluble flavonoids and polyphenols [69]. *S. zenkeri* was most effective in its hydroethanolic extract (0.2 *μ*g/mL), followed by aqueous and ethanolic forms, reflecting its dense phenolic matrix [80]. *S. aromaticum* exhibited moderate ABTS activity, with the hydroethanolic extract (0.3 *μ*g/mL) outperforming aqueous and ethanolic forms. Some IC_50_ values were extremely low; while they may reflect strong radical scavenging under assay conditions, they should be interpreted cautiously, as DPPH and ABTS assays are sensitive to matrix effects and do not predict physiological efficacy [81, 82]. Factors such as bioavailability and metabolism further modulate antioxidant relevance in vivo [83].

In the FRAP assay, which measures reducing power through electron‐donating capacity, all extracts showed higher EC_50_ values than vitamin C (18 *μ*g/mL). Among the spices, *S. zenkeri* stood out, with its aqueous extract showing the strongest effect (29 *μ*g/mL), consistent with its high polyphenol and tannin content [80]. *S. aromaticum* also demonstrated notable reducing power, particularly in its aqueous and ethanolic extracts (48–302 *μ*g/mL), in agreement with the electron‐donating properties of eugenol and gallic acid derivatives. *X. aethiopica* displayed moderate reducing capacity, with the aqueous extract (416 *μ*g/mL) outperforming other solvent fractions, a trend previously associated with alkaloids, terpenoids, and flavonoids [66]. In contrast, *M. whitei* exhibited the weakest FRAP response, although its aqueous extract (684 *μ*g/mL) surpassed its hydroethanolic and ethanolic forms. Despite its relatively high phenolic content, the antioxidant profile of *M. whitei* appears to rely more on radical scavenging than on direct electron transfer, as suggested in earlier studies [69].

Taken together, the DPPH, ABTS, and FRAP results illustrate how extraction polarity shapes the antioxidant profiles of the four spices, with each assay highlighting different facets of their electron‐donating and radical‐scavenging behavior under in vitro conditions.

### 3.5. Anti‐Inflammatory Activity

The anti‐inflammatory potential of *M. whitei*, *S. zenkeri*, *S. aromaticum*, and *X. aethiopica* was evaluated through inhibition of COX‐1, COX‐2, and 5‐LOX (Table 4), enzymes central to prostaglandin and leukotriene biosynthesis and relevant to vascular inflammation and endothelial dysfunction. The extracts displayed clear interspecies and solvent‐dependent variations, reflecting differences in extract composition and polarity. As these are cell‐free biochemical assays, the activities reported here represent preliminary indicators of inhibitory potential.

**Table 4:** IC_50_ values (micrograms per milliliter) for anti‐inflammatory activity of spice extracts.

Assay	Extraction type	*Mondia whitei* (roots)	*Scorodophloeus zenkeri* (bark)	*Syzygium aromaticum* (flower bud)	*Xylopia aethiopica* (seeds)	Reference (indomethacin)
COX‐1	Aqueous	22 ± 2ᵇᵞ	24 ± 3ᵇᵞ	20 ± 2ᵃᵝ	27 ± 3ᶜᵞ	12 ± 2
	Ethanolic	18 ± 2ᵇᵝ	19 ± 2ᵇᵝ	16 ± 2ᵃᵝ	20 ± 2ᶜᵝ	
	Hydroethanolic (30/70)	15 ± 2ᵃᵅ	17 ± 2ᵇᵅ	15 ± 2ᵃᵅ	19 ± 2ᶜᵅ	
COX‐2	Aqueous	112 ± 10ᵇᵞ	117 ± 10ᶜᵞ	109 ± 9ᵃᵞ	121 ± 11ᵈᵞ	100 ± 10
	Ethanolic	13 ± 2ᵇᵝ	14 ± 2ᵇᵝ	12 ± 2ᵃᵝ	16 ± 2ᶜᵝ	
	Hydroethanolic (30/70)	11 ± 2ᵃᵅ	11 ± 2ᵃᵅ	10 ± 1ᵃᵅ	12 ± 2ᵇᵅ	
5‐LOX	Aqueous	1010 ± 90ᵇᵞ	1100 ± 100ᵈᵞ	995 ± 85ᵃᵞ	1070 ± 100ᶜᵞ	1000 ± 90
	Ethanolic	36 ± 4ᵇᵝ	37 ± 4ᵇᵝ	28.4 ± 3ᵃᵝ	40 ± 4ᶜᵝ	
	Hydroethanolic (30/70)	23 ± 3ᵇᵅ	27 ± 3ᵇᵅ	21 ± 3ᵃᵅ	26 ± 3ᵇᵅ	

*Note:* Values are expressed as mean ± SD (*n* = 3). A two‐way ANOVA (factors: species × solvent) followed by Tukey′s post hoc test was performed. For each solvent, different lowercase superscript letters (a–d) indicate significant differences among species (*p* < 0.05). For each species, different Greek superscript letters (*α*–*γ*) indicate significant differences among solvents (*p* < 0.05). Lowercase and Greek superscripts represent independent comparison sets and should not be compared across rows or columns.

Abbreviations: COX, cyclooxygenase; IC_50_, half‐maximal inhibitory; LOX, lipoxygenase.

In the COX‐1 assay, aqueous extracts of all four spices showed weaker inhibition than indomethacin (12 *μ*g/mL), with IC_50_ values ranging from 20 to 27 *μ*g/mL. Ethanolic and hydroethanolic extracts demonstrated lower IC_50_ values (15–20 *μ*g/mL), approaching the reference inhibitor under the same conditions. Among them, hydroethanolic extracts of *M. whitei* and *S. aromaticum* (15 *μ*g/mL) were statistically comparable to indomethacin, indicating similar inhibitory behavior within this assay format. These observations are consistent with earlier reports describing the contribution of flavonoid‐ and polyphenol‐rich matrices to COX‐1 modulation [84]. The moderate inhibition observed here is noteworthy, as partial COX‐1 suppression may attenuate inflammation while preserving mucosal protection.

In the COX‐2 assay, aqueous extracts were less effective than indomethacin (112–121 *μ*g/mL vs. 100 *μ*g/mL). In contrast, ethanolic and hydroethanolic extracts showed markedly lower IC_50_ values (10–16 *μ*g/mL), indicating strong inhibition under assay conditions. The hydroethanolic extract of *M. whitei* (11 *μ*g/mL) was among the most active, consistent with previous observations linking its phenolic and flavonoid content to COX‐2 modulation [85]. *S. aromaticum* also showed strong COX‐2 inhibition across organic extracts (10–12 *μ*g/mL), coherent with the anti‐inflammatory properties attributed to eugenol, gallic acid derivatives, and flavonoid aglycones. Selective COX‐2 inhibition is particularly relevant in cardiometabolic disorders, where chronic low‐grade inflammation contributes to insulin resistance and vascular dysfunction.

In the 5‐LOX assay, aqueous extracts showed IC_50_ values comparable to indomethacin (≈1000–1100 *μ*g/mL). Ethanolic and hydroethanolic extracts exhibited much lower IC_50_ values (21–40 *μ*g/mL), indicating strong inhibition under assay conditions. The hydroethanolic extract of *M. whitei* again showed the lowest value (23 *μ*g/mL), while *S. aromaticum* also demonstrated strong activity (21–28 *μ*g/mL). These results align with earlier reports describing the leukotriene‐modulating properties of phenolic‐rich extracts [84]. Although these IC_50_ values are much lower than those of indomethacin in this assay, they should be interpreted cautiously, as crude extracts may influence absorbance‐based detection or background signal.

Taken together, the COX‐1, COX‐2, and 5‐LOX results illustrate how extraction polarity shapes the anti‐inflammatory profiles of the four spices, with organic and mixed solvents generally enhancing inhibitory performance under in vitro conditions.

### 3.6. Phytochemical–Bioactivity Correlations and Solvent Effects

**Figure 2:**
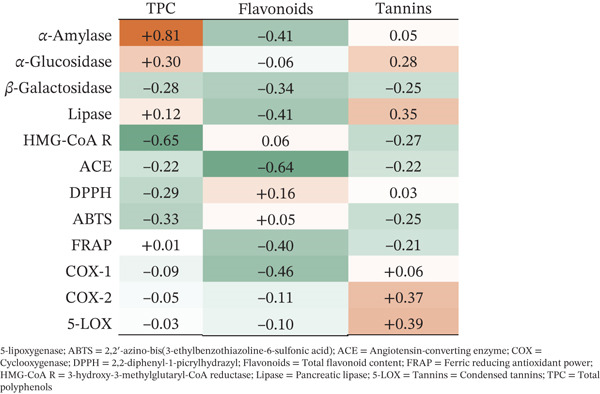
Integrated Pearson′s correlation matrix and corresponding heat map illustrating relationships among phytochemical constituents, antioxidant assays, and cardiometabolic/inflammatory enzyme‐related endpoints in spice extracts. Color intensity represents correlation strength and direction; significant correlations (*p* < 0.05) are indicated. 5‐LOX, 5 lipoxygenase; ABTS, 2,2 ^′^‐azino‐bis(3 ethylbenzothiazoline 6 sulfonic acid); ACE, angiotensin‐converting enzyme; COX, cyclooxygenase; DPPH, 2,2‐diphenyl‐1‐picrylhydrazyl; flavonoids, total flavonoid content; FRAP, ferric reducing antioxidant power; HMG‐CoA R, 3‐hydroxy‐3‐methylglutaryl‐CoA reductase; lipase, pancreatic lipase; 5‐LOX, tannins = condensed tannins; TPC, total polyphenols.

The correlation analysis (Figure 2) revealed heterogeneous and assay‐specific relationships between TPCs, flavonoids, tannins, and the biological endpoints evaluated in this study, underscoring the complexity of phytochemical–bioactivity interactions in spice extracts. When interpreted alongside the phytochemical and functional data presented in Sections 3.2–3.5, these patterns confirm that extracts exhibiting strong inhibitory responses, such as *α*‐amylase (*r* up to 0.81) or ACE (*r* down to −0.64), do not necessarily correspond to those with the highest levels of total phenolics or flavonoids. Instead, the matrix highlights selective associations between particular phytochemical classes and specific enzyme targets, suggesting that biological potency depends more on the structural diversity and reactivity of individual phenolic subclasses than on bulk phytochemical content. Antioxidant assays showed weaker or inconsistent correlations (generally |*r*| < 0.35), a trend consistent with previous reports indicating that radical‐scavenging responses are often driven by highly reactive phenolics, synergistic interactions, or nonphenolic antioxidants rather than total phenolic load [86, 87]. Similarly, weak correlations between total phenolics and antioxidant assays have been documented in several spice and medicinal plant studies, reflecting the multifactorial nature of antioxidant mechanisms and the contribution of diverse chemical families [88]. This multifactorial behavior, where processing conditions shape multifunctional outcomes, is not unique to phytochemistry. Comparable principles have been reported in polymer science, where cellulose nanocrystals improve the multifunctional properties of PLA–graphene/carbon nanotube composites by modulating dispersion and processability [89]. Such cross‐disciplinary parallels reinforce the idea that extraction polarity and material processing jointly determine functional signatures.

**Figure 3:**
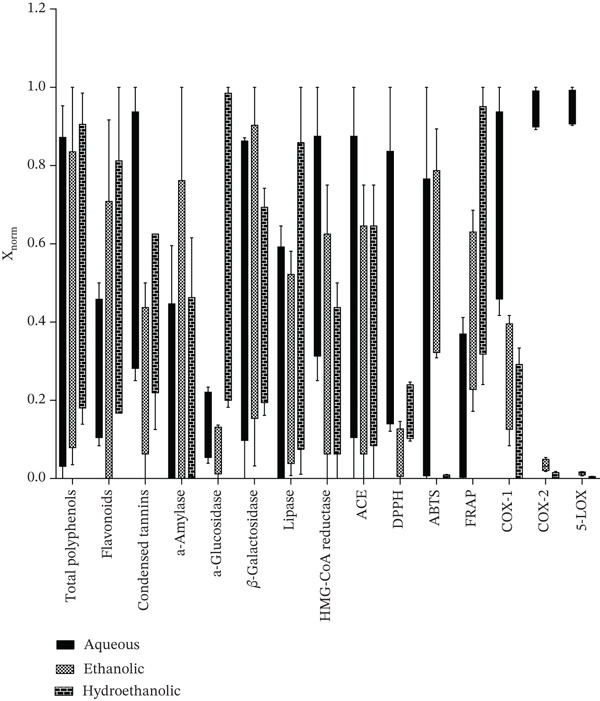
Comparative boxplot of min–max normalized phytocomposition and biological activities across extraction solvents.

When examined alongside the solvent‐dependent normalized profiles (Figure 3), these correlations help contextualize how extraction polarity shapes both phytochemical recovery and biological performance. For phytochemical content, hydroethanolic extracts showed the highest normalized levels of TPCs (0.52) and flavonoids (0.34), whereas aqueous extracts displayed the highest condensed tannin content (0.59). Ethanolic extracts consistently exhibited lower normalized values for these constituents, reflecting reduced extraction of hydrophilic and semipolar phenolics.

For cardiometabolic enzymes, the normalized values revealed clear solvent‐dependent differences in biological activity, with lower values corresponding to stronger inhibition. Among carbohydrate‐related enzymes, *α*‐amylase showed comparable activity across solvents (0.15–0.26), whereas *α*‐glucosidase inhibition was strongest in ethanolic extracts (0.08) and weakest in hydroethanolic extracts (0.59). *β*‐Galactosidase activity varied only slightly between solvents (0.44–0.54), and lipase inhibition was strongest in ethanolic (0.27) and aqueous (0.27) extracts, whereas hydroethanolic extracts showed weaker inhibition (0.43). These patterns indicate that ethanol and water preferentially recover compounds acting on carbohydrate‐ and lipid‐processing pathways. For cholesterol‐ and blood pressure–related enzymes, aqueous extracts displayed the weakest inhibition (highest normalized values) for HMG‐CoA reductase (0.56) and ACE (0.48), whereas hydroethanolic extracts showed the strongest inhibition (lowest normalized values: 0.25 and 0.35, respectively). Ethanolic extracts exhibited intermediate activity. This distribution suggests that hydroethanol more effectively extracts compounds modulating cholesterol biosynthesis and vascular regulation.

Antioxidant activities also followed solvent‐dependent trends. Stronger DPPH scavenging corresponded to lower normalized values, which were observed in ethanolic (0.06) and hydroethanolic (0.17) extracts, whereas aqueous extracts showed weaker activity (0.42). ABTS inhibition was strongest in hydroethanolic extracts (0.01), followed by aqueous (0.27), while ethanolic extracts showed weaker activity (0.51). FRAP reducing power was strongest in aqueous (0.17) and ethanolic (0.43) extracts, whereas hydroethanolic extracts showed weaker activity (0.65). These complementary patterns highlight that each solvent emphasizes a different facet of antioxidant performance.

For inflammatory enzymes, the lowest normalized values, and therefore the strongest inhibition, were observed in hydroethanolic (COX‐1: 0.13; COX‐2: 0.01; 5‐LOX: 0.00) and ethanolic extracts (COX‐1: 0.27; COX‐2: 0.03; 5‐LOX: 0.01). Aqueous extracts showed very high normalized values (0.69–0.95), indicating weak inhibition of COX‐1, COX‐2, and 5‐LOX. This contrast demonstrates that hydroethanol and ethanol preferentially extract compounds acting on eicosanoid‐related inflammatory pathways.

Taken together, the correlation patterns (Figure 2) and solvent‐dependent profiles (Figure 3) show that extraction polarity governs both phytochemical composition and biological activity. The correlation trends highlight the exploratory nature of the dataset and the need for deeper chemical profiling to identify the constituents responsible for the multifunctional activities observed. As polarity increases from ethanol → hydroethanol → water, phytochemical recovery shifts accordingly: Hydroethanol enriches semipolar phenolics, whereas water concentrates condensed tannins. For cardiometabolic enzymes, ethanolic and hydroethanolic extracts consistently produced lower normalized values and thus stronger inhibition for *α*‐glucosidase, lipase, HMG‐CoA reductase, and ACE, indicating that less polar solvents better extract compounds acting on carbohydrate digestion, lipid processing, cholesterol biosynthesis, and vascular regulation. Antioxidant responses also followed polarity‐dependent trends, with aqueous extracts showing weaker activity and ethanolic/hydroethanolic extracts displaying stronger DPPH, ABTS, and FRAP performance. Inflammatory enzymes showed the clearest contrast, with hydroethanolic and ethanolic extracts producing the strongest inhibition and aqueous extracts the weakest. Overall, these observations confirm that solvent polarity is a key determinant of extract functionality and reinforce the multifactorial nature of spice bioactivity.

## 4. Conclusion

This study provides preliminary insight into the phytochemical composition and in vitro antioxidant, metabolic, and anti‐inflammatory activities of *S. zenkeri*, *M. whitei*, *S. aromaticum*, and *X. aethiopica*. The four species exhibited distinct activity profiles reflecting differences in phenolic, flavonoid, and tannin content, and the correlation analysis showed that these phytochemical classes were selectively associated with specific biological endpoints rather than uniformly predictive of activity. Solvent polarity further modulated these relationships: Hydroethanolic extraction enhanced the recovery of semipolar phenolics and improved several activities in *M. whitei*, *S. zenkeri*, and *S. aromaticum*, whereas ethanolic extraction was more effective for *X. aethiopica*. Together, these findings indicate that both phytochemical–bioactivity correlations and solvent polarity shape the functional signatures of the extracts. Although the results suggest promising bioactive potential, this remains exploratory and requires comprehensive chemical characterization, deeper profiling of active constituents, evaluation in cellular systems, toxicity assessment, and in vivo validation to clarify mechanisms of action and support future integration of these indigenous Cameroonian spices into functional food, nutraceutical, or agroindustrial value chains.

## Author Contributions

**Kendra Lucrèce Nkaleu Ngonganga:** investigation, methodology, writing – original draft, writing – review and editing. **Achille Nouga Bissoue:** conceptualization, writing – review and editing, visualization, supervision. **Blanche Cunégonde Omgba Etoundi:** writing – review and editing, visualization. **Aymar Rodrigue Fogang Mba:** writing – review and editing, visualization. **Ahlam Ali:** writing – review and editing, visualization. **Roberta Tolve:** writing – review and editing, formal analysis, data curation, visualization. **Fideline Laure Tchuenbou-Magaia:** writing – review and editing, visualization, supervision. **Fabrice Fabien Dongho Dongmo:** conceptualization, investigation, writing – original draft, writing – review and editing, supervision.

## Funding

No funding was received for this manuscript.

## Disclosure

The study was conducted as part of the institutional employment of the authors at the University of Douala (Cameroon), the University of Wolverhampton (United Kingdom), and the University of Verona (Italy).

## Ethics Statement

Thisz study did not involve human participants or animals, and therefore did not require ethical approval.

## Conflicts of Interest

The authors declare no conflicts of interest.

## Data Availability

The data supporting the findings of this study are available from the corresponding author upon reasonable request.
